# Professional Notes

**Published:** 1887-10-08

**Authors:** 


### Professional Notes.

By a Physician.

Coffee powder, Dr. Offler considers the best substance for disguising the odour of iodoform. The coffee should be freshly roasted and thoroughly pulverised in a mortar. The powder is said to possess mild antiseptic properties.

Black eyes and bruises in other prominent parts of the person are very inconvenient and annoying. The strong tincture of capsicum, applied with gum, is said to act as an effective decolorant.

Newly produced burns are intensely painful, and drive some people almost frantic. Dr. Zuboff regards permanganate of potash as an efficacious remedy for the pain and inflammation of the acute stage. Compresses thoroughly soaked and frequently changed give early and permanent relief.

The uses of massage are various. Dr. Murrell considers that it is of service in the obstinate constipation of women. It has obtained some reputation also as a reducer of fat. In both these disorders it is well worthy of a trial; and probably some modified method which could easily be carried out at home would be sufficient if regularly persevered with.

In an address on Clothing, delivered at the annual meeting of the British Medical Association, Mr. Charles Moore Jessop affirms that "a human being well clothed, well housed, and well fed, is more sensitive to atmospheric and other changes than one in an opposite condition, whose senses are blunted, not by privation but from want of cultivation. The skin and brain, or central nervous system, are in embryological relationship. When both systems are fully developed in the human animal capable of shifting for itself, the more highly developed portion takes cognisance of its peripheral or outer layer, the skin, according to surrounding circumstances. If the skin be unclothed, sensations, both internal and external, are obtuse. . . . But if clothed, the skin is rendered more sensitive to atmospherical vicissitudes, and central sensitiveness is exalted."

"The Parisian fashion," says Mr. Jessop "of accentuating, *more Romanorum*, that portion of the chest which should merge into the waist is not for the advantage of the *sex*, because it is a custom fertile in disease and death. The back, shoulders, and arms, with half the bosom exposed, is nakedness without modesty. It is not beautiful, for the witchery of dress is absent. Duplicate hollows, prominences, and angularities detract from that assemblage of properties which attracts and pleases the eye; the impression of oneness is lost. . . . The half-clothed Indian is picturesque, not the completely naked. . . . The upper half of an old Indian man or woman is positively repulsive."

Sleeplessness is on the increase, especially among busy and worried men. It makes the patient impatient if he is told that the best cure is to lessen his worries and take more outdoor exercise. He has neither self-control to accomplish the former, nor physical strength to enjoy the latter. Much, therefore, as the practice of procuring artificial sleep is to be deprecated as a rule, it is sometimes the only possible course open to the physician. It is then for him to choose that method of procedure which shall procure the required sleep with the smallest possible measure of physiological injury in other directions. Dr. Dujardin-Beaumetz considers paraldehyde one of the safest and most effectual hypnotics we possess. It usually agrees well with the stomach, and does not depress the heart; on the contrary, the pulse gains in strength under its action. In the insomnia caused by alcoholism it is said to be superior to chloral. In other cerebral affections, as noisy dementia, convulsive neuroses, and all the innumerable forms of hysteria, it is exceedingly useful. When chloral is, for any reason, contra-indicated. the paraldehyde may usually be resorted to. As an antidote in strychnia poisoning it is highly spoken of. The dose is from half a drachm to two drachms, given with diluted syrup and almond mixture.

"Health in man," says Dr. Lauder Brunton, "as in other animals, depends upon the proper performance of all the functions. These functions may be shortly said to be three: (1) Tissue change; (2) Removal of waste; (3) Supply of new material. For the activity of man, like the heat of the fire by which he cooks his food, is maintained by combustion; and just as the fire may be prevented from burning brightly by improper disposition of the fuel, or imperfect supply of air, and as it will certainly go out if fresh fuel be not supplied, and may be choked by its own ashes; so man's activity may be lessened by imperfect tissue-change, and may be put an end to by an insufficient supply of new material, and imperfect removal of waste products."

"A mixture of foods is best adapted to supply the energy and repair the waste in the human body. Like the steam-engine, we require oils and fats; proteids which go, in a great measure at least, to repair the wear and tear of the tissues; and carbo-hydrates, which may be looked upon as like coal, supplying energy to the organism by their combustion. These various kinds of food are required in different proportions. According to Ranke, about 100 grammes of proteids and a similar quantity of fats are required daily by a man, while two-and-a-half times as much, that is 250 grammes, of carbo-hydrates are necessary."

Diseases of the joints are among the most troublesome the family practitioner has to deal with; not necessarily from want of knowledge and skill on his part. Perfect rest is as a rule absolutely essential, and this the patient often has not the sense to see, and the doctor has not the courage to insist upon. Hence arise frequent disagreements between attendant and patient, and sometimes an entire destruction of that mutual confidence which is the primary condition of successful family practice. On this important point Professor Annandale says: "Perfect rest, and a proper position of the joint; the application of soothing means locally in acute disease, of counter-irritants and pressure in chronic disease, according to the nature of the affection, and the employment of constitutional treatment if required"—these are essential. "In order to insure perfect rest and a proper position of the joint, elastic and other bandages, splints made of leather, pasteboard, or of more substantial material, as the 'MacIntyre,' or a modification of it, are found most useful. . . . In some painful forms of joint-disease in the hip or knee, rest, position, and a separation of the affected articular surfaces of the joint, can be most successfully obtained by the employment of a weight, or other apparatus, so as to extend the limb. When any joint-disease is causing constitutional irritation dangerous to life, the propriety of excision or amputation must be considered, and the operation most suitable in the particular case at once performed." To experienced practitioners, these observations are perfectly trite; but they are like the ten commandments, always true, and always needing to be firmly impressed upon the professional and lay mind.

